# “…we have to think first what we are going to feed our children before we have them …”: Rwandan women use family planning to provide a better life for their children

**DOI:** 10.1371/journal.pone.0246132

**Published:** 2021-04-22

**Authors:** Hilary M. Schwandt, Angel Boulware, Julia Corey, Ana Herrera, Ethan Hudler, Claudette Imbabazi, Ilia King, Jessica Linus, Innocent Manzi, Madelyn Merrit, Lyn Mezier, Abigail Miller, Haley Morris, Dieudonne Musemakweli, Uwase Musekura, Divine Mutuyimana, Chimene Ntakarutimana, Nirali Patel, Adriana Scanteianu, Biganette-Evidente Shemeza, Gi’anna Sterling-Donaldson, Chantal Umutoni, Lyse Uwera, Madeleine Zeiler, Seth Feinberg

**Affiliations:** 1 Fairhaven College, Western Washington University, Bellingham, Washington, United States of America; 2 Spelman College, Atlanta, Georgia, United States of America; 3 Wheaton College, Norton, Massachusetts, United States of America; 4 Northwest Vista Community College, San Antonio, Texas, United States of America; 5 Whatcom Community College, Bellingham, Washington, United States of America; 6 INES, Ruhengeri, Musanze, Rwanda; 7 Xavier University, New Orleans, Louisiana, United States of America; 8 UMBC, Baltimore, Maryland, United States of America; 9 Department of Sociology, Western Washington University, Bellingham, Washington, United States of America; 10 SUNY Oswego, Oswego, New York, United States of America; 11 Western Oregon University, Monmouth, Oregon, United States of America; 12 Eastern Oregon University, La Grande, Oregon, United States of America; 13 University of Kentucky, Lexington, Kentucky, United States of America; 14 Arcadia University, Glenside, Pennsylvania, United States of America; 15 Rutgers, New Brunswick, New Jersey, United States of America; 16 Drexel University, Philadelphia, Pennsylvania, United States of America; Hackensack Meridian Health, UNITED STATES

## Abstract

Use of modern contraception in Rwanda has risen dramatically over a short time period. To better understand contraceptive users’ motivations for family planning services in Rwanda, 32 in-depth interviews with contraceptive users and eight focus groups with 88 family planning providers were conducted in Rwanda’s Musanze and Nyamasheke districts. Study participants noted how family planning is critical for providing a better life for children. Family planning gives mothers independence from childcare to work in order to provide for their children’s wellbeing. Family planning presented an opportunity for generational upward mobility and was perceived as a way to contribute positively to society.

## Introduction

Meeting the global need for family planning and, therefore, averting unwanted pregnancies would result in significant reductions in maternal and newborn deaths, unsafe abortions, and disability [1]. There are an array of motivations for family planning use. The motivation, however, that is often most prominent for individuals considering contraceptive use for themselves, is financial. Studies with men in Kenya, Malawi, and Togo found that the men associated family planning with the financial advantages of smaller family size [2–4]. While both men and women in Uganda gave economic reasons for using family planning, this motivation resonated more strongly with men [5]. Women in rural Ethiopia explained how their contraceptive use gave them more time to pursue work outside the home to contribute to family expenses [6]. The most commonly cited reason for using contraception among men and women in Rwanda, among both contraceptives users and nonusers, was the finances needed to take care of children [7]. Individuals citing financial motivations for family planning use often describe how family planning enables them to cover child related expenses, such as education, food, and clothing [4, 5, 7].

Rwanda’s contraceptive prevalence rate dramatically increased at the beginning of the century from 17% to 52% between 2005–2015 [8]; much of this increase has been among women who are living in rural areas and those with less education [9]. During this same time period, the poverty level concurrently dropped from 59% in 2001 to 39% in 2014 [8]. With relative peace and stability, along with aggressive development goals outlined by the central government, economic growth in Rwanda has increased on average 7.5% per year between 2008–2018, with GDP per capita rising 5% annually prior to the global COVID-19 impacts (World Bank 2020). Family planning is viewed by the current government of Rwanda as an important component of broader economic development initiatives [10, 11].

Rwanda’s unique family planning program success can be attributed to top-down support from the highest levels of Rwandan governance, including consistent messaging and promotion of family planning benefits expressed from high-ranking government officials [12–15]. The government of Rwanda has also worked to decentralize the health care system, establish a national health insurance program, improve and expand infrastructure, institute performance-based incentives, as well as implement and support the Community Health Worker (CHW) program [10, 14, 16, 17]. This support is linked to constitutional reforms to promote gender equity with the recognition that women’s empowerment is good for individual women, their families, as well as the nation overall.

The promotion of family planning for national economic development is clearly articulated via policy. Given the existing literature on the linkages between family planning use and financial considerations in the region, this study aimed to understand motivations for family planning use in Rwanda from experienced modern contraceptive users and family planning providers in Rwanda. This study expands upon the existing literature by including the perspectives of family planning providers on motivations for family planning, and elucidating the motivations for and benefits from contraceptive use among female Rwandan contraceptive users—for themselves, their children, and their society, as well as operationalizing the pathway from contraceptive use to financial security for women.

## Methods

This study was conducted between February and July of 2018. Narrative data were collected via focus group discussions and in-depth interviews in the Musanze and Nyamasheke districts of Rwanda. These represent the districts of the country with the highest and lowest rates of modern contraceptive prevalence rates, respectively [8]. The eight focus group discussions were conducted with family planning providers: four with Community Health Workers (CHW) and four with family planning nurses. Each FGD had between eight and 12 participants. A total of 88 family planning providers participated in the FGDs. The 32 interviews were conducted with experienced modern contraceptive users.

Family planning providers were recruited by local Rwandan NGO employees, government hospital-based staff, and administrators. Family planning provider recruiters were instructed to recruit CHWs who provide family planning and nurses whose primary role is to provide family planning who were 18 years of age and older. Modern contraceptive users were recruited by family planning providers. Modern contraceptive recruiters were instructed to find female modern contraceptive users who had used modern contraception for at least six months and women who had discontinued modern contraceptive use within the last six months, for reasons other than desiring pregnancy, who were 18 years and older.

The sample size was determined prior to data collection based upon guidance in the literature to elicit information from at least two focus groups per characteristic, in this case there were two characteristics: provider type and district, and at least eight in-depth interviews per characteristic, in this case there were also two: current modern contraceptive use and district [18]. The study design initially included eight modern contraceptive users and another eight who had discontinued a modern contraceptive method, in each district. Post study participant recruitment and initiation of data collection, it was noted that a misunderstanding occurred between the researchers and the recruiters in terms of what constituted discontinuation of modern contraception—as well as difficulty in finding recent discontinuers. Due to the timing of this discovery after recruitment was significantly underway, the study plans were permanently modified, and no attempt was made at this late stage of recruitment to initiate recruitment of recent discontinuers. As a result, the contraceptive users included in this study were all experienced modern contraceptive users, and the vast majority were currently using modern contraception.

All of the focus groups and interviews were conducted in Kinyarwanda. The focus group discussions averaged two hours in length and the interviews approximately 50 minutes each. The study participants and the research team did not have any relationships with each other prior to data collection. The focus group discussions were conducted in private meeting rooms. Individuals were interviewed in mutually agreeable locations. The focus group discussions and in-depth interviews were conducted by one interviewer during the interviews and a team of two during the focus groups, a moderator and a note taker.

The topic guides were developed based upon experience with topic guide development in previous, similar research studies, prior research among stakeholders in the same setting [12], and review of existing literature on family planning in Rwanda. The FGD topic guide included a storytelling activity designed to elicit norms and motivations for contraceptive use in various scenarios—as well as provider thoughts and responses to those clients, a risk-ranking activity to understand views about risks of various reproductive health scenarios in comparison to each other, as well as general questions about working in family planning and advice based on those experiences. The interview topic guide included questions under the following theme headings: contraceptive use experience; experience accessing contraceptives; motivations, social networks, and fears; and giving advice to others. Researchers used the topic guides to elicit descriptive information from the study participants—they were not read verbatim. The translated topic guides were piloted and pre-tested in Kigali prior to initiating data collection in the field. The topic guides remained unchanged throughout data collection.

All data collection activities were recorded using digital recorders upon permission of study participants. The audio recordings were translated and transcribed into English. Data analysis was guided by the thematic content analysis approach [19] and executed using Atlas.ti 8 software [20]. The research team developed codes from reading and re-reading the translated transcripts. Each team member coded the same transcript independently, and then collectively shared codes and coding patterns with the entire research team. The team then developed an agreed upon code list, with definitions, to guide subsequent coding of the remaining transcripts. Inter-rater reliability was not assessed as part of the process.

Once coding of all transcripts was complete, individual or group level matrices were executed for each code using Microsoft Excel, where analysis was done within code, looking for further subthemes, patterns, differences by characteristics, and illustrative quotes. Data were analyzed by district and characteristics. Differences by characteristic were only noted in instances when the themes, comments, or quantity of responses differed by district or sampled characteristic. When the analysis showed no, or little, difference by district or sampled characteristic, this is not noted.

Approval from the Institutional Review Boards at the Rwandan Ministry of Education in Kigali, Rwanda and Western Washington University in Bellingham, Washington occurred prior to data collection in Rwanda, and all study participants signed informed consent documents prior to participation in the study. Study participants were compensated 10,000 Rwandan francs for participation to accommodate them for their time and to cover transportation costs (~$10 USD at the time of data collection).

## Results

### Study participant demographics

Among the 88 family planning providers who participated in this study, 52% were located in Musanze and 54% were CHWs. The average age of the providers was 44 and ranged between 28 and 61. Most of the providers were female, at 73%. Providers had worked on average 6 years, ranging between 1 year and 22 years. The providers on average had a parity of four, ranging from one to nine. Most providers, 91%, had experience with family planning use. The most commonly used method by providers was implant, at 35% of the sample, followed by injectable, 31%, and sterilization 13%.

The average age of the 32 experienced modern contraceptive users was 38, ranging from 26 to 50. Average parity was four, ranging from one to nine. Most women, at 81%, were farmers. The most common method currently, or most recently, used was injectable at 41% of the sample, followed by condoms, 22%, and the implant, 19%.

### Themes

The primary theme that arose in this study was the motivation to use family planning to increase family financial security. When asked about motivation for family planning use, every respondent highlighted this central theme. Respondents shared their perspectives on how the use of family planning methods allowed women to space and limit births, freeing them to work and make money to help develop, or manage, the family—with a primary focus on financial provision to meet the children’s needs. Additional subthemes include a woman’s desire to provide multiple needs and long-term benefits for children, to strengthen and empower her own physical and financial health, and to contribute to the overall development of the nation.

### Family financial security

Contraceptive users, when asked about motivations to use family planning, linked their decisions to use family planning with their household income.

I: Why don’t you want other children?

R: We don’t have more money.

Female, 50, injectable user, 5 children, Musanze, IDI

…we have to look at our finances and have children based on our finances. And if you don’t have that much income, you decide to not have as many children.

Female, 31, injectable user, 4 children, Musanze, IDI

Having kids is like running a business because you have to plan when you will have them and how you will raise them. That is why we should use family planning.

Female, 48, injectable user, 6 children, Musanze, IDI

Family planning providers also discussed financial considerations of family size as a primary factor for clients’ decision-making—considerations of the family’s ability to manage, or have the financial means to provide for, all expenses related to the children and family as a unit.

…the number of children that she wants to have is taken into her own hands based on her finances.

Nurse, female, Musanze, FGD

You should not give birth to children that you do not have enough money for or can’t support.

CHW, male, 46, Musanze, FGD

Couples use family planning to achieve their desired spacing and limiting needs to only have the number of children that they can adequately provide for. Women often used the phrase, having the number of children they had the “ability to care for,” in relation to reasons for using family planning. Providing a good life for children meant having the means to financially meet their needs.

…I am proud of having children that I am able to care for.

Female, 32, pill user, 3 children, Musanze, IDI

…if I did not go to use family planning by now I would have already had like five children very close in age that I wouldn’t be able to care for.

Female, 31, injectable user, 3 children, Musanze, IDI

Family planning providers also discussed the motivation to use family planning to have fewer demands and a greater ability to care for children.

I think the reason why she wanted to use family planning is because she wanted to have children that she is able to raise and she wanted to give her children the care that they deserve.

Nurse, female, 36, Nyamasheke, FGD

Most of the benefits of family planning stemmed from having better financial status due to the ability to work as a result of spacing and limiting the number of children—respondents often referred to life after initiation of family planning as a “better life”.

I saw that contraceptive use was important because I saw that having more kids was not beneficial, because if I have a small number of kids that I can take care of it will give me peace, and will also give me a better life.

Female, 41, injectable user, 5 children, Musanze, IDI

…right now if you don’t work hard it will be difficult to have a better future for your kids.

Female, 41, condom user, 5 children, Musanze, IDI

The theme of family planning use leading to a better life/good life, or the ability to live well, also arose in nearly every focus group discussion with family planning providers.

When she will start to use family planning methods she will see that she is capable to have the number of children she wants and also when she wants. She will see that her life will be better…

Nurse, female, 38, Nyamasheke, FGD

The reason why she has thought to use family planning is in order to give her children a better life.

Nurse, female, 34, Musanze, FGD

### Children’s wellbeing

Women were motivated to use family planning to be able to provide for their children’s basic necessities. Respondents often said that they wanted to achieve a standard of living beyond mere sustenance and be able to fund school fees as well as provide food and clothing. Women demonstrated that their decision to use family planning and limit the number of children they had took into consideration the long-term finances of the family and provisions for any possible future children.

That is the main reason that led me to take that decision (using family planning) so that I can take care of my child and raise him and provide basic needs. When your child asks you for something and you cannot provide it—that breaks your heart.

Female, 26, injectable user, 1 child, Musanze, IDI

#### Food and nutrition

Providing food for children arose often as a subtheme in provision of basic necessities.

…we have to think first what we are going to feed our children before we have them.

Female, 32, pill user, 3 children, Musanze, IDI

…you don’t always have stress in thinking about what you are going to feed your children because you have children that you are able to raise.

Female, 34, pill user, 2 children, Musanze, IDI

Family planning providers also noted the financial burden related to the ability to afford food for the children.

When you don’t use family planning you can have too many kids to care for based on your financial circumstance, they may face malnutrition.

CHW, female, 45, Nyamasheke, FGD

#### Health insurance

The topic of paying for health insurance for the entire family arose as both a motivator to use family planning as well as an economic reason for using family planning. Study participants noted how health insurance is a new fiscal responsibility for citizens (in the last 10 years). While the very poor are still offered free health care, the new norm is that families pay for health insurance for their families at a unit price per each member of the household, hence, the more children one has in their family the more expensive the health insurance bill is annually. For those who have health insurance, family planning is provided for free.

…when you have few children and also have to pay Mutual Health it becomes easier for you. Imagine like me, now I pay 15,000 as we are five people in the family. If I add another child, it might be 18,000, so this cannot be easier for me because I am increasing the expenses but our income is still the same. And then it becomes a burden to me.

Female, 32, pill user, 3 children, Musanze, IDI

…some who have children close in age will miss out on health insurance because they don’t have much money—just kids without money.

Female, 50, injectable user, 8 children, Musanze, IDI

#### Providing for multiple necessities

Study participants often discussed the difficulty in providing more than one necessity for their children in the same response.

Children need shoes, clothes, and notebooks for school. Life is complicated, that’s why I don’t want any more children.

Female, 48, injectable user, 6 children, Musanze, IDI

…right now I have two boys and one girl, with not much property to give to my boys, and it costs a lot of money to pay health insurance. It is not easy to find clothes for my kids to wear, and it is difficult to meet the nutrition needs of my children. So I told myself that I can’t have more children than I am able to raise.

Female, 38, pill user, 3 children, Musanze, IDI

She thought about school, she thought about insurance…she thought about development, she thought about feeding her child, because if a child feeds good, they grow up well.

CHW, female, 45, Nyamasheke, FGD

#### Schooling

The vast majority of women interviewed made mention of school fees for children as a motivator for and benefit of using family planning. It was common for women to note that limiting the number of children they had allowed them to save money and ensure that all of their children were able to attend school. This theme arose more often among Nyamasheke participants as compared to participants from Musanze. Family planning providers contributed to this theme as well.

Maybe she is looking towards her future and she sees that if she has many children, she will not be able to educate them well…

Nurse, female, 54, Nyamasheke, FGD

If they use a family planning method, it will help them to prepare a good future for their children. They will have enough time to save for the education of their children.

Nurse, female, 44, Nyamasheke, FGD

#### Societal benefits

Women also showed that they were not only motivated to limit the number of children they had for the betterment of their own family, but for the betterment of society as well. They wanted to be able to pay for the school fees of their children so that the country as a whole could progress in development with a more educated population and so their children would be able to provide for themselves as adults, instead of being a burden to society.

When you are using family planning you are also helping the country in the way of development for the country. If we are sending our children to school, they become good leaders for the country.

Female, 29, implant user, 2 children, Nyamasheke, IDI

Family planning providers also discussed the cascading effect of using family planning on increased education of citizens and the growth of the nation.

She thought about the good of having children on her own terms. The good thing about that is that she is able to take care of them on her own and for her own benefit and the benefit of the country.

CHW, male, 43, Musanze, FGD

It was also common for women to frame the benefits their family experienced on a broader, societal scale. A relatively common subtheme was women noting that without family planning, they would not be able to provide for all the children they would have had and so some of their children would have had to resort to stealing. In this context, they said family planning was not only good for the well-being of the family, but also for society because it prevented children from becoming thieves.

I don’t want my children to be on the road begging and being a burden to others so that’s why I decided to start using family planning.

Female, 38, injectable user, 5 children, Musanze, IDI

The reason why I don’t want more kids is because having kids is a good thing, but to not be able to give them what they need is a hard thing for me. That is why, for myself, I decided that I should stop giving birth…if you continue to have more kids and you aren’t able to provide for their needs, then your kids might have to steal to get what they need.

Female, 35, condom user, 4 children, Nyamasheke, IDI

### Woman’s independence, strength, and appearance

#### Woman’s independence to work

Within this subtheme study participants described the ease of life when not encumbered with caring for young children. Individual women mentioned this theme on multiple occasions in the same interview, highlighting the impact of this theme in being both a motivation for use of family planning and a benefit gained from it. Women often noted the need to work to help provide for the needs of the family—and how the use of family planning allowed women to work, whereas taking care of young, and many closely spaced children would prevent her from doing so.

I: What benefits have you gained when you compare the time you hadn’t started using contraceptives and after you started?

R: I gained many things. Before I was staying home all of the time, because the first priority that I had was to take care of my children. If you want to do other things that can improve your life you couldn’t do that because you had a responsibility first to take care of the children. When I started using contraceptives things became easier and I gained many things because there were no obstacles that stopped me from what I wanted to do. Now I can explore all the things that will help my family grow stronger.

Female, 41, injectable user, 5 children, Musanze, IDI

Nearly half of the interviewees mentioned a benefit of family planning use as being “baby free”, all of them referenced the ease of being able to find and do work and earn money for their family when not burdened with the care of an infant. Being free from carrying a baby on your back, as is traditionally done, facilitates the agrarian work that most women interviewed do. Respondents reported that they could not even find a place of employment while they had a young baby, let alone, being able to do the work.

…if you carry a baby on your back no one can give you a job…I am strong now and if I want a job I can go and search for it. Nothing holds me back.

Female, 38, pill user, 3 children, Musanze, IDI

Before, I could not even manage to buy myself clothes because I was always carrying a baby so I could not find any jobs. But then I started to use family planning, I started being able to buy myself and my children clothes, and food for my children.

Female, 32, injectable user, 3 children, Musanze, IDI

Family planning providers also contributed to the theme of being baby free in terms of freedom to work and contribute to the household finances.

By the time she wants to use family planning she’s able to work for her house and her family. Because you see when she gives birth to kids who are close in age it’s tiring because she has one on the back and one in hand and that is very tiring.

CHW, female, 60, Musanze, FGD

#### Women’s strength to work

Better health, and therefore strength, for women was also noted as a benefit of family planning use. Women frequently reported that being able to control birth spacing and having more time to take care of themselves following childbirth allowed them to have better health. The ultimate thrust of having better health, and improved strength, was the ability to work and make money to continue to support the family needs.

If you have kids close in age you won’t have the strength to work because you often spend your time caring for the babies and the other tasks that need to be done at home. This causes the development of your family to decrease.

Female, 45, pill user, 2 children, Nyamasheke, IDI

At the time I started to use family planning, I had more energy to work for my family and earn more money with my husband. And now I have two children who are in high school and I am able to pay for their school fees. And life is good now.

Female, 43, injectable user, 4 children, Nyamasheke, IDI

…when I had two kids and wasn’t using contraceptives, the age between the first born and the second born the age was one year, but when I started using contraceptives the age between the other births I had was five years, and I benefited from that because I gave birth when I was strong and that’s helped me to improve not only myself but my family, too.

Female, 41, condom user, 5 children, Musanze, IDI

#### Preventing premature aging

Respondents also cited a desire to prevent premature aging as an impetus for contraceptive use. This theme arose more often among Musanze contraceptive users as compared to contraceptive users from Nyamasheke.

Another reason that motivated me to use family planning was that at that time I was having children that were close in age and because of that, I was looking like an old woman while I was still young in age. And I asked myself, "Why do people always say that I look older than I am?". It was due to having children close in age and always being pregnant and so I decided to use family planning and I returned to my normal appearance (laughs).

Female, 43, injectable user, 4 children, Nyamasheke, IDI

You can see that contraceptives changed something within me, and I am becoming more youthful again.

Female, 38, pill user, 3 children, Musanze, IDI

Women who referenced aging in terms of appearance were often motivated by wanting to look younger. An interesting subtheme here was how looking younger was thought to help ensure a husband’s fidelity.

And the person who has more kids looks different because she looks older. It’s a problem because when you see the men of those women, they sleep around because the woman is not that pretty. The men go to look for pretty girls.

Female, 42, injectable user, 3 children, Musanze, IDI

We take care of ourselves, because when you are not taking care of yourself your husband can go looking for other women.

Female, 29, implant user, 2 children, Nyamasheke, IDI

## Discussion

When asked about motivations for using family planning, every theme related to the woman’s ability to work outside the home to make money to support the development of their family—primarily focused on the wellbeing of her existing children. Family planning allows mother’s employment, with employment comes financial gains, and with fewer children comes cost reductions. As others conclude as well, fewer children enables enhanced parental investment in the wellbeing of their children [3, 21, 22]. Prior research on the attitudes of men in sub-Saharan Africa has shown that financial gains are their main motivation for family planning use as well [2–4]. This finding has also been replicated among women in other settings [5, 6, 23]; therefore, it would behoove family planning programs to focus on the financial benefits of family planning use as it resonates with both females and males.

In this study, participants discussed the financial benefits from family planning use not only for themselves and their families, but for the wider society as well. The political leadership around family planning [11], likely contributed to the linkages study participants made between their individual and family decisions and their impact on the development of the broader society.

Women using family planning had entered into a positive feedback loop where the motivation to use family planning—to have the money necessary to provide for their children’s wellbeing—were also the benefits gained from family planning, which sustained their motivation to seek out and use family planning when desiring to space or limit births, while sexually active and fecund, and able to access the appropriate services and methods. In sub-Saharan Africa where fertility remains high, as do associated negative health outcomes, an important area of focus is understanding how women enter into and have the necessary support and access to remain in this positive feedback loop when sexually active, fecund, and not desiring pregnancy.

The tipping point of entry into this positive feedback loop appeared to be primarily the experience of short birth intervals with initial births, or fear of the same, which motivated the use of family planning to elongate future birth intervals or limit births. Researchers have studied the impact of power and equity on contraceptive use and have found there is a threshold effect [24], suggesting that for this tipping point to work, a threshold of equity with male partners is necessary. Past research in Rwanda has shown that strong political leadership from the top-down promoting the national family planning program in Rwanda has contributed to a number of successes, potentially leading to equity for some in reproductive health decision-making [12].

In this study women voiced their motivations for family planning use and the benefits gained from it. Women are using family planning to improve the lives of their children, but their own selves seem to be absent from the motivations and benefits of use. Even in terms of the woman’s health—it was noted in terms of the ability to have the strength to work to provide for her children. The only time women discussed contraceptive use benefiting themselves was in terms of avoiding rapid aging; however, for some this was quickly linked to maintaining the fidelity of their husband—which is a fear likely also linked to financial repercussions.

This study has a number of strengths. Family planning providers as well as current family planning users were both included in the study. The use of qualitative methods allowed for a deeper understanding behind the motivations for family planning use and the subsequent benefits derived. Despite these strengths, the study does suffer from some limitations. Non-contraceptive users were not included in the study, nor were male partners. There was also a misunderstanding between the study team and the participant recruiters for the individual interviews so recent discontinuers were not included in the sample as originally designed. In addition, women were referred to our study through family planning providers, so it is likely that women sampled had positive experiences with family planning. The topic guide and questions asked were biased toward positive experiences, while negative experiences were included and probed, they were not done so with equal coverage. Finally, it would have been better had transcription occurred first, prior to translation, as opposed to happening in the same step for translation accuracy.

In conclusion, this study found that family financial security is the main motivation for contraceptive use in Rwanda. Women used contraception to create space in between births to give them the freedom and strength to work for pay outside the home in order to contribute to the children’s expenses, primarily: school fees, health insurance, and food. Their efforts to support their children were seen to benefit not only their children and their families, but the wider society as well. Women in this study identified entering into a positive feedback loop, where the benefits gained from contraceptive use supported the motivations for continuing to use contraception. In this context, it is easy to envision a landscape of continued, and sustained contraception use by current users, and the effects spilling over into additional users in the near future with the potential for broad familial and societal benefits.

## Supporting information

S1 File FP providers FGD topic guide. (DOCX)

S2 File Kinyarwanda FP providers FGD topic guide. (DOCX)

S3 File FP users topic guide. (DOCX)

S4 File Kinyarwanda FP users topic guide. (DOCX)
