# Supplementary material for: “…we have to think first what we are going to feed our children before we have them …”: Rwandan women use family planning to provide a better life for their children
Source: PLoS One. 2021 Apr 22;16(4):e0246132. doi: 10.1371/journal.pone.0246132 (PMC8062032; doi:10.1371/journal.pone.0246132)
Supplement: S1 File — (DOCX) [file pone.0246132.s001.docx]

**Family Planning Providers in Rwanda**

**Focus Group Discussion Topic Guide**

| **ACTIVITY 1 : STORY TELLING** |
| --- |
| **Suggested time:** About 20 minutes.  **Materials**: story prompts  ***Note to Facilitator:***  *In this activity, you will read the story prompts and ask participants to engage in a discussion after the story.* |

**Story, part one:**

Claudette is 25 years old and married to Benjamin, 30 years. They have 2 girls, the oldest is 4 and the youngest is 1 and a half years. Claudette would like to delay her next birth. She thinks oral contraceptive pills will be her best option.

**Discussion prompts:**

1. What has motivated Claudette to think about using family planning?
2. Who has the most influence over deciding when and how many children Claudette should have? Why?
3. What will she do next?
4. Who will she discuss her decision with?
5. Describe the experience she will have when seeking out family planning methods.
   - How do the family planning providers respond to Claudette’s needs?
   - Describe factors that affect the services that Claudette receives when she seeks out family planning.
6. Describe how the staff respond to Claudette’s request for a specific method.

**Story, part 2:**

Two months later, Claudette decides to stop using the pills due to side effects. She seeks out a provider to help her find a better method.

**Discussion prompts:**

1. Why do you think Claudette stopped using the pill?
2. Describe the interaction that occurs between Claudette and her provider when she goes back to the clinic.
3. Does she leave with the same contraceptive method or a different method or no method?

**Story, part 3:**

Claudette has a younger sister, Nellie. Nellie is unmarried and pregnant. She is interested in using family planning after the birth of her baby.

**Discussion prompts:**

1. Who does Nellie go to for family planning services?
2. Describe how she is received by those she goes to for help.

**Story, part 4:**

Claudette and Nellie have a younger sister, Alice. She is 20 years old and is sexually active with her boyfriend. Since Nellie got pregnant, Alice is more attuned to her own risks of pregnancy. She would like to use family planning to avoid pregnancy.

**Discussion prompts:**

1. Who does Alice go to for family planning services?
2. Describe how she is received by those she goes to for help.

| **ACTIVITY 2: GENERAL QUESTIONS** |
| --- |
| **Suggested time:** About 20 minutes.  **Materials**: None  ***Note to Facilitator:***  *In this activity, you will read the question prompts and ask participants to discuss the questions.* |

General Questions about Providing Family Planning Services

1. Describe the typical family planning client.
2. Describe the easy aspects of being a family planning provider.
3. Describe the difficult aspects of this job.
4. What aspects of this job have gotten easier with the increase in family planning use in the nation? What has gotten more difficult?
5. What would have to change to make this job better?
6. What motivates family planning providers to be the best they can be for family planning clients? What factors detract from this motivation?
7. How could family planning providers change to make the experience of accessing the services better for the clients?

| **ACTIVITY 3: RISK PERCEPTION (Technique: Card-ranking; Discussion)** |
| --- |
| **Suggested time:** About 20 minutes.  **Materials**: 3 risk cards (Most risky, somewhat risky, least risky)  action cards  Tape (to tape card to wall) or stones (to hold cards firm on ground)  ***Note to Facilitator:***  *In this activity, you will ask participants to rank a set of actions based on the level of risk they think each action poses to health. Participants will place each action card in order from least risky to most risky. Try to get participants to all agree on the order of the cards. If this is not possible, you can use the majority opinion to make the final decision.*  *This is a good opportunity to energize the group after a lot of discussion so make sure all the participants are involved and moving around to place / move the action cards on the ground or floor.*  *At the end of the activity, ensure the notetaker makes a record of the final ranking.* |

**Step 1:** Place 3 cards on the ground or on the wall – at the far left place “Least risky”, in the middle “somewhat risky”, and at the far right “most risky”.

**Step 2:** One by one, ask participants to place the following picture cards in order from least risky to most risky, guiding discussion for each card using the following prompts. Continue discussion until the group agrees on the order of the cards. Each action should be more risky than the action to its left.

Discussion prompts:

- Why is this action risky?
- Why is ‘x’ more/less risky than ‘y’?

Action cards:

- Oral contraceptive pills
- Condoms
- Sterilization
- Abortion
- Implants
- IUD
- injectables
- Getting pregnant soon after having a baby
- Having a birth under 18 years of age
- Having 6 children
- fertility awareness methods
- Getting pregnant outside of marriage

| **ACTIVITY 4: GIVING ADVICE** |
| --- |
| **Suggested time:** About 20 minutes.  **Materials**: none  ***Note to Facilitator:***  *In this activity, you will read the question prompts and ask participants to discuss the questions.* |

1. What advice might you have for Rwandans interested in using family planning methods for the first time?
2. What advice might you have for Rwandans considering discontinuing family planning use?
3. What advice might you have for fellow family planning service providers in Rwanda?
4. What advice might you have for improving the family planning program in Rwanda?
5. What advice might you have for others working in family planning program in other countries, where family planning use is lower than it is in Rwanda?
