# Supplementary material for: “…we have to think first what we are going to feed our children before we have them …”: Rwandan women use family planning to provide a better life for their children
Source: PLoS One. 2021 Apr 22;16(4):e0246132. doi: 10.1371/journal.pone.0246132 (PMC8062032; doi:10.1371/journal.pone.0246132)
Supplement: S2 File — (DOCX) [file pone.0246132.s002.docx]

**ABAFATANYABIKORWA KUBIJYANYE NO KUBONEZA URUBYARO MU RWANDA**

**IKIGENDERWAKO MUBIGANIRO BYO MW’ ITSINDA**

| **IGIKORWA CYA 1:KUBARA INKURU**  **Umwanya uteganyijw**e: iminota 20  **Ibikoresho :** inkuru y’integuza  **Icyitonderwa  k’umufasha** :  Muri iki gikorwa turasoma inkuru nteguza maze tubaze abajyanama bubuzima kugira icyo bavuga nyuma y’inkuru. |
| --- |

**INKURU, IGICE CYA MBERE :**

Korodeta afite imyaka 25 akaba yarashakanye na Benyamini w’ imyaka mirongo 30. Bafite abakobwa 2, umukuru afite imyaka 4, umutoya afite umwaka umwe n’igicye.Korodeta ashaka gukurikiza bitinze .Yatekerejeko ibinini aribwo buryo bwiza bwamufasha kuboneza urubyaro.

**IHANGIRO RY’ IKIGANIRO :**

1. Niki cyatumye Korodeta atekereza kuboneza urubyaro ?
2. Ninde wagize uruhare runini ku cyemezo cya Korodeta, Korodeta yagakwiye kugira abana bangahe kd ryari  ? kubera iki ?
3. N’iki azakora nyuma ?
4. Ninde azaganiriza  icyemezo yafashe ?
5. Garagaza ubumenyi azagira mugihe azaba akoresha uburyo bwo kuboneza urubyaro ?

- Nigute abajyanama b’ubuzima mu kuboneza urubyaro bazafasha Korodeta mubyo yifuza ?
- Ni izihe mbogamizi Korodeta azahura nazo mugihe azajya kwaka serivise yo kuboneza urubyaro .

6. garagaza ukuntu ubuyobozi bw’ ikigo nderabuzima buzafasha Korodeta muguhitamo inzira nziza yo kuboneza urubyaro.

**INKURU, IGICYE CYA KABIRI :**

Hashize amezi abiri, Korodeta afata icyemezo cyo guhagarika gukoresha ibinini bitewe n’ingaruka byamutgizeho. Yahise ashaka umukangurambaga kugirango amufashe guhitamo ubundi buryo bwiza.

**IHANGIRO RY’ ikiganiro :**

1. Nigiki watekereza cyatumye korodeta ahagarika gukoresha ibinini ?
2. Garagaza ikiganiro kizaba hagati ya Korodeta n’umukangurambaga mugihe azasubira ku ivuriro.
3. Azahava atahanye bwa buryo yakoreshaga mbere, azahindura cg azarekera gukoresha uburyo bwo kuboneza urubyaro ?

**INKURU IGICYE CYA 3 :**

Korodeta afite murumuna we NELLIE. Nellie ntiyubatse ariko aratwite. Arifuza kuboneza urubyaro namara kubyara.

**IHANGIRO RY’ IKIGANIRO :**

1. Ninde Nellie yagana k’ ubufasha bwo kuboneza urubyaro ?
2. Garagaza uburyo azakirwa n’abo azagana agiye kwaka ubufasha.

**INKURU, IGICYE CYA 4 :**

Korodeta na Nellie bafite murumuna wabo Alice . Alice afite imyaka 20 kandi akora imibonano mpuza bitsina numukunzi we. Kuva Nellie yatwita, Alice afite ubwoba ko yatwita.Yifuza gukoresha uburyo bwo kuboneza urubyaro mu kwirinda gusama.

**IHANGIRO RY’ IKIGANIRO:**

1. Ninde Alice yagana k’ ubufasha bwo kuboneza urubyaro ?
2. Garagaza uburyo azakirwa n’abo azagana agiye kwaka ubufasha.

| **IGIKORWA CYAKABIRI: IBIBAZO NYAMUKURU** |
| --- |
| **Umwanya utegekanijwe:** iminota 20  **Ibikoresho :** zero  **Icyitonderwa k’ umufatanyabikorwa**  Muriki gikorwa, uzasoma ihangiro ry’ ibibazo wongere abahashitse gutanga intererano kubibazo |

**IBIBAZO RUSANGE KUBIJANYE NO KUBONEZA URUBYARO**

1. Garagaza amatsinda y’ abantu bakoresha gahunda yo kuboneza urubyaro.
2. Garagaza ibiranga umujyanama w’ubuzima mu kuboneza urubyaro.
3. Garagaza imbogamizi nyamukuru z’aka kazi .
4. Ni ibihe bimenyetso byerekana ko aka kazi kagiye koroha bijyanye nuko abakoresha gahunda yo kuboneza urubyaro bagiye biyongera mu gihugu. Ni iki cyabaye imbogamizi?
5. Ni iki cyahindurwa mu buryo bwo gutuma akazi karushaho kuba keza?
6. Ni iki gitera abajyanama bubuzima mubyo kuboneza urubyaro bakorera neza ababagana?Ni ibiki byatuma imikorere yabo ihinduka?(iba mibi)
7. Nigute abajyanama b’ubuzima mu kuboneza urubyaro bashobora kworohereza ababagana.

| **IGIKORWA CYA GATATU: ukwigaragaza kw’ ingaruka ( ubuhanga : gutondekanya amakarita: ikiganiro)** |
| --- |
| **Umwanya utegekanyijwe:** iminota 20  **Ibikoresho:** amakarita atatu (ingaruka ikomeye, ingaruka iringaniye, ingaruka yoroheje)  Madika ikarita k’ urukuta cyangwa ku ibuye kugira zoye kunyiganyiga  **Icyitonderwa k’ umufatanya bikorwa:**  Muri kino gikorwa, muzasaba abitabiriye gutondeka ikarata bagendeye ku uburemere bw’ ingaruka zashobora kubangamira ubuzima. Abitabiriye bazotondeka amakarita kurutonde bahereye kuyoroheje ujya ku ikomeye.mugerageze kumvisha abitabiriye uko urutonde rw’amakarita rukurikirana.ibyo bidashobotse ,wagendera kugitekerezo cy’abenshi mugufata icyemezo.  Ubu ni uburyo bwiza bwogusubizamo imbaraga itsinda nyuma y’ ibiganiro mukerekezo cyo kumenyako abitabiriye bashizemo imbaraga mukumadika ikarita k’urukuta. K’ umusozo w’ igikorwa ,genzura neza ko wufata umwanzuro yakoze urutonde rwiza. |

**Intambwe ya mbere:** shyira ikarita eshatu ku butaka cyangwa k’ urukuta , kure ibumoso muhashyire ikarita y’ingaruka zoroheje,hagati ingaruka ziringaniye hanyuma kure iburyo harajya ibifite ingaruka nyinshi.

**Intambwe ya kabiri:** umwe kuri umwe, musabe abahageze gutondeka amakarita k’ urutonde uhereye kuyoroheje ukageza ku ikomeye, uyobore ikiganiro mu ntego zikurikira. Komeza ikiganiro kugeza aho itsinda ryose rihuriza k’ urutonde rw’ ikarita.

**IHANGIRO RY’ IKIGANIRO**

- Ni kuki iki gikorwa gifite imbogamizi?
- Ni kuki intanga ngabo ifite imbogamizi nke ku ntanga ngore
- Ibinini byo kunywa
- agakingirizo
- kwifungisha burundu
- gukuramo inda
- agapira
- urunigi
- Urushyinge
- Gutwita mu gihe gito ubyaye.
- Kubyara umwana musi y’ imyaka 18
- Kubyara abana batandatu
- Kumenya igihe nyacyo cyo gutwita
- Gutwita waciye inyuma umugabo

| **IGIKORWA CYA KANE : GUTANGA INAMA** |
| --- |
| **Umwanya utegekanijwe :** iminota20  **Ibikoresho :** zero  **Icyitonderwa k’ umufatanyabikorwa :**  Muri kino gikorwa, muzasoma intego y’ ikibazo hama mubaze abahashitse gutanga ibiterezo |

23. Ni iyihe nama mwagira abanyarwanda bakoresha uburyo bwo kuboneza urubyaro ku ncuro ya mbere ?

24. Ni iyihe nama mwagira abanyarwanda bagambiriye kureka kuboneza urubyaro ?

25. Ni iyihe nama mwagira incuti zanyu babajyanama bubuzima mu kuboneza urubyaro mu Rwanda ?

26. Ni iyihe nama mwatanga igamije kuzamura gahunda yo kuboneza urubyaro mu Rwanda ?

27. Ni iyihe nama mwagira abandi bakora mur gahunda yo kuboneza urubyaro mubindi bihugu aho ikoreshwa rya gahunda yo kuboneza urubyaro rikiri hasi ugereranije no mu Rwanda ?
