# Supplementary material for: “…we have to think first what we are going to feed our children before we have them …”: Rwandan women use family planning to provide a better life for their children
Source: PLoS One. 2021 Apr 22;16(4):e0246132. doi: 10.1371/journal.pone.0246132 (PMC8062032; doi:10.1371/journal.pone.0246132)
Supplement: S3 File — (DOCX) [file pone.0246132.s003.docx]

**Family Planning Users in Rwanda**

**In-Depth Interview Topic Guide**

Demographic Questions

1. What type of work do you do?
2. How old are you?
3. Are you married? Or living with your partner?
4. Do you have any children? If yes, how many? What are their ages?
5. Do you want to have more children? If yes, how soon would you like to get pregnant?

Contraceptive Use Questions

1. Can you tell me the names of all the contraceptive methods you are aware of?
2. What type of contraceptive method are you currently using?
3. How long have you been using this method? For how much longer do you plan on using this method?
4. Can you tell me about your experiences using this method?
5. Do you have any plans to switch to another method in the future?
6. Can you describe why you chose this method over the other options available?
7. Have you tried any other types of contraceptive methods? If yes, which ones have you tried? Can you tell me about your experience with these methods? Can you describe why you chose these methods?

Service Delivery Questions

1. Can you tell me about your experiences accessing the method you are using now?
2. Can you describe your experiences with the family planning providers?

Motivations, Social Networks, and Fears

1. Can you describe to me why you decided to start using family planning?
2. Can you tell me how your partner felt about this decision? Your parents? Other family members? Your friends?
3. Can you explain to me how you first learned about family planning?
4. Can you describe to me if and how you and your friends discuss family planning?
5. Can you describe your biggest fears as they related to contraceptive use?

Giving Advice

1. What advice might you have for others interested in using family planning methods?
2. What advice might you have for family planning service providers in Rwanda?
3. What advice might you have for improving the family planning program in Rwanda?
