# Supplementary material for: “…we have to think first what we are going to feed our children before we have them …”: Rwandan women use family planning to provide a better life for their children
Source: PLoS One. 2021 Apr 22;16(4):e0246132. doi: 10.1371/journal.pone.0246132 (PMC8062032; doi:10.1371/journal.pone.0246132)
Supplement: S4 File — (DOCX) [file pone.0246132.s004.docx]

**ABAKORESHYA UBURYO BWOKUBONEZA URUBYARO MU RWANDA**.

**IKIGANIRO KIRAMBUYE**

**IBIBAZO KUBYEREKEYE UMWIRONDORO**

1. Mukora Uwuhe murimo?
2. Mufite imyaka ingahe?
3. Murubatse? Mubana n’ umugabo/umugore?
4. Mufite abana? Niba mubafite ni bangahe? Bafite imyaka ingahe ?
5. Mwaba mwifuza abandi bana ? Niba ari yego, mwaba mwifuza gusama ryari ?

**IBIBAZO BIJYANYE N’UBURYO BWO KUBONEZA URUBYARO**

6. Mwatubwira uburyo mwaba muzi bukoreshwa mu kuboneza urubyaro ?

7. Ni ubuhe buryo mukunda gukoresha ?

8. Mumaze igihe kingana gute mukoresha ubu buryo? Mwifuza gukomeza kubukoresha mugihe kingana gute ?

9.Mwaba mufite ubumenyi mugukoresha ubu buryo ?

10. Mwaba muteganya guhindura uburyo bwokuboneza urubyaro ?

11. Niyihe mpamvu yatumye muhitamo gukoresha buno buryo ?

12. Hari ubundi buryo mwaba mwaragerageje ? Niba ari yego, mwagerageje ubuhe buryo ? Ni ubuhe bumenyi mufite kuri ubwo buryo mwagerageje? Kuki mwahisemo gukoresha ubwo buryo ?

**IBIBAZO KU ITANGWA RYA SERIVISI**

13. Mwambwira uko mugezwaho serivisi ugendeye kuburyo bwo kuboneza urubyaro mukoresha ?

14. Nubuhe bumenyi mufite mugukorana n’abakangurambaga muri gahunda yo ukuboneza urubyaro ?

**UMWETE (motivation), IMBUGA NKORANYAMBAGA, HAMWE N’ IMBOGAMIZI**

15. Mwatubwira impamvu zatumye mufata icyemezo cyo kuboneza urubyaro ?

16. Ni gute umufasha wawe yakiriye iki cyemezo ? Ababyeyi bawe ?Abandi bantu bo mu muryango ? Inshuti zawe ?

17. Ni gute mwamenye ububuryo bwokuboneza urubyaro ?

18. Ese wowe ninshuti zawe mujya muganira ibyerekeranye no kuboneza urubyaro ?niba mubiganira mubiganira mute ?

19. Ni izihe mbogamizi nyamukuru muhuranazo mu byerekeranye no kuboneza urubyaro ?

**GUTANGA INAMA**

20. Ni iyihe nama watanga kubantu bifuza gukoresha uburyo bwo kuboneza urubyaro ?

21. Ni iyihe nama watanga kubakangurambaga kubijyanye no kuboneza urubyaro mu Rwanda ?

22. Ni iyihe nama watanga kubyerekeranye no kongera imikorere kuri gahunda yo kuboneza urubyaro ?
